# Single-photon detection in the mid-infrared up to 10 *μ*m wavelength using tungsten silicide superconducting nanowire detectors

**DOI:** 10.1063/5.0048049

**Published:** 2021

**Authors:** V. B. Verma, B. Korzh, A. B. Walter, A. E. Lita, R. M. Briggs, M. Colangelo, Y. Zhai, E. E. Wollman, A. D. Beyer, J. P. Allmaras, H. Vora, D. Zhu, E. Schmidt, A. G. Kozorezov, K. K. Berggren, R. P. Mirin, S. W. Nam, M. D. Shaw

**Affiliations:** 1 National Institute of Standards and Technology, Boulder, Colorado 80305, USA; 2 Jet Propulsion Laboratory, California Institute of Technology, 4800 Oak Grove Dr., Pasadena, California 91109, USA; 3 Department of Electrical Engineering and Computer Science, Massachusetts Institute of Technology, Cambridge, Massachusetts 02139, USA; 4 Department of Physics, Lancaster University, Lancaster, United Kingdom

## Abstract

We developed superconducting nanowire single-photon detectors based on tungsten silicide, which show saturated internal detection efficiency up to a wavelength of 10 *μ*m. These detectors are promising for applications in the mid-infrared requiring sub-nanosecond timing, ultra-high gain stability, low dark counts, and high efficiency, such as chemical sensing, LIDAR, dark matter searches, and exoplanet spectroscopy.

## I. INTRODUCTION

Efficient single-photon counting, with a detection efficiency greater than 50%, has, to date, been achieved only at wavelengths shorter than 2 *μ*m.^1^ Extension of such performance to the mid-infrared has potential for new applications in astronomy,^2^ as well as LIDAR,^3^ dark matter searches,^4^ and the fundamental study of fast molecular dynamics and chemistry.^5–8^ Interest in the mid-infrared stems primarily from the presence of numerous absorption signatures for molecules such as water vapor, carbon dioxide, oxygen and ozone, methane, and nitrous oxide. These molecules not only are important for understanding the evolution of our own planet but may also be signatures of life or indicate the potential habitability of planets outside of our solar system. The spectroscopy of exoplanet atmospheres is a prime example of an application requiring single-photon-counting detectors with ultra-stable gain and high efficiency due to the photon-starved nature of the measurements.^9,10^

Currently, the most common detector technology in the mid-infrared wavelength range is based on the semiconductor HgCdTe. Small-format HgCdTe avalanche photodiode (APD) arrays have become available, which are single-photon sensitive and can operate at a temperature of 77 K or higher.^11^ Another common technology, which has been used extensively in astronomy, is the blocked impurity band detector (BIB). These are silicon-based detectors that are not single-photon sensitive but, nevertheless, have demonstrated high quantum yield (>60%) over a broad range of wavelengths spanning from 4 to 28 *μ*m.^12^ BIB detector arrays are not as common as HgCdTe arrays due to the fact that they operate at a lower temperature of ~10 K.

Superconducting single-photon detectors have also shown promise in the mid-infrared and in general have the advantages of significantly lower dark count rates and improved gain stability when compared with semiconductor-based detectors. In particular, arrays of microwave kinetic inductance detectors (MKIDs) have been widely used in astronomy due to their large array formats and are being explored for use in the mid-infrared.^13^ Transition edge sensors (TESs) have also been considered as a detector technology, which may be useful for exoplanet spectroscopy although, to date, there has not been much experimental work in optimizing them for the mid-infrared.^14^ Both of these technologies operate at extremely low temperatures, typically on the order of 0.1 K.

Superconducting nanowire single-photon detectors (SNSPDs) are another class of superconducting detector, which can operate at relatively high temperatures compared to MKIDs and TESs, typically between 1 and 4 K. SNSPDs have been used extensively in the quantum optics field due to their high efficiency (98% at 1550 nm),^15^ larger dynamic range, higher count rates compared to MKIDs and TESs and intrinsic dark count rates of less than 1 count/day.^4^ Arrays as large as 1 kilopixel have also been demonstrated, enabling a wide range of new potential applications.^16^ Although previous work has focused primarily on telecommunication wavelengths, recently there has been significant interest in using these detectors in the mid-infrared. In particular, the low jitter and high dynamic range make SNSPDs ideal for space-to-ground or fiber-based quantum key distribution and LIDAR.^17^ Low noise and extremely stable gain make SNSPDs ideal for exoplanet spectroscopy.^2^

Here, we report on SNSPDs based on tungsten silicide (WSi) with saturated internal detection efficiency up to a wavelength of 10 *μ*m. Internal detection efficiency is defined as the probability that a photon absorbed in the nanowire generates an electrical output pulse from the detector. Prior work in the near-infrared has shown that achieving saturated internal detection efficiency is a critical step in making high efficiency devices.^15,18^ Our results indicate that it may be possible to achieve high system detection efficiency in the mid-infrared with WSi SNSPDs by optimizing the absorption in the nanowires and the coupling to the active area of the detectors, as has been done at near-infrared wavelengths.

Previous approaches to achieving sensitivity in the mid-infrared were based on SNSPDs fabricated from niobium nitride (NbN) thin films.^19,20^ NbN has historically been the material of choice for SNSPDs due to its relatively high superconducting transition temperature (8–12 K) allowing operation at 4 K and its fast thermal recovery time allowing operation at high count rates.^21^ In order to improve the energy sensitivity of the nanowire detectors and extend the photo-response to longer wavelengths, there are several possible approaches.

The first approach is to decrease the cross-sectional area of the nanowire, making the film thinner or the nanowire narrower. This results in a higher probability of a hotspot (non-superconducting domain) being generated since the energy per unit area is larger, and thermal conduction along the length of the nanowire is smaller. Using this approach, saturated internal detection efficiency has been demonstrated with NbN SNSPDs up to a wavelength of ~3 *μ*m using ultra-narrow 30 nm-wide nanowires.^19^ In another report, single-photon sensitivity was achieved at a wavelength of 10.6 *μ*m using 40 nm-wide NbN nanowires although the internal detection efficiency was not saturated.^20^

The second approach to improving energy sensitivity is to engineer the material to reduce the free carrier density. Reducing the free carrier density (increasing resistivity) of the material results in the deposited energy being divided among fewer quasiparticles, thus increasing their effective temperature. Simultaneously, the thermal impedance along the length of the nanowire is increased, which more effectively localizes the deposited energy along its length. This approach allows the width and thickness of the nanowires to be larger, which is better from a fabrication and yield perspective since the maximum achievable fraction of the depairing current has been shown to decrease for narrower nanowires.^22^ As the nanowire becomes narrower, edge roughness and constrictions in the width of the nanowire, due to fabrication imperfections, begin to have a larger relative effect and degrade the device performance. The constrictions result in a suppressed switching current, the current at which the critical current density in the wire is exceeded and the superconductor switches to the normal (non-superconducting) state. Since the minimum detectable photon energy depends on the fraction of the depairing current that the detector is biased at,^23–25^ it is important to minimize the impact of edge roughness and constrictions. It is expected that the edge roughness is on the order of 5 nm, limited by the electron-beam resist processing, which is good enough to yield nanowires down to 50 nm without significant degradation in performance.

A third approach to improving energy sensitivity is reducing the superconducting gap energy. This results in a larger number of broken Cooper pairs (quasiparticles) for a given amount of deposited energy in the superconductor and a larger fraction of photon energy accumulated in the electronic system.^26^ However, reducing the superconducting gap energy implies a lower superconducting transition temperature (*T*_c_) and thus a lower operating temperature for the detectors. This is generally undesirable as it results in an increase in the complexity and cost of the cryogenics.

**FIG. 1.**
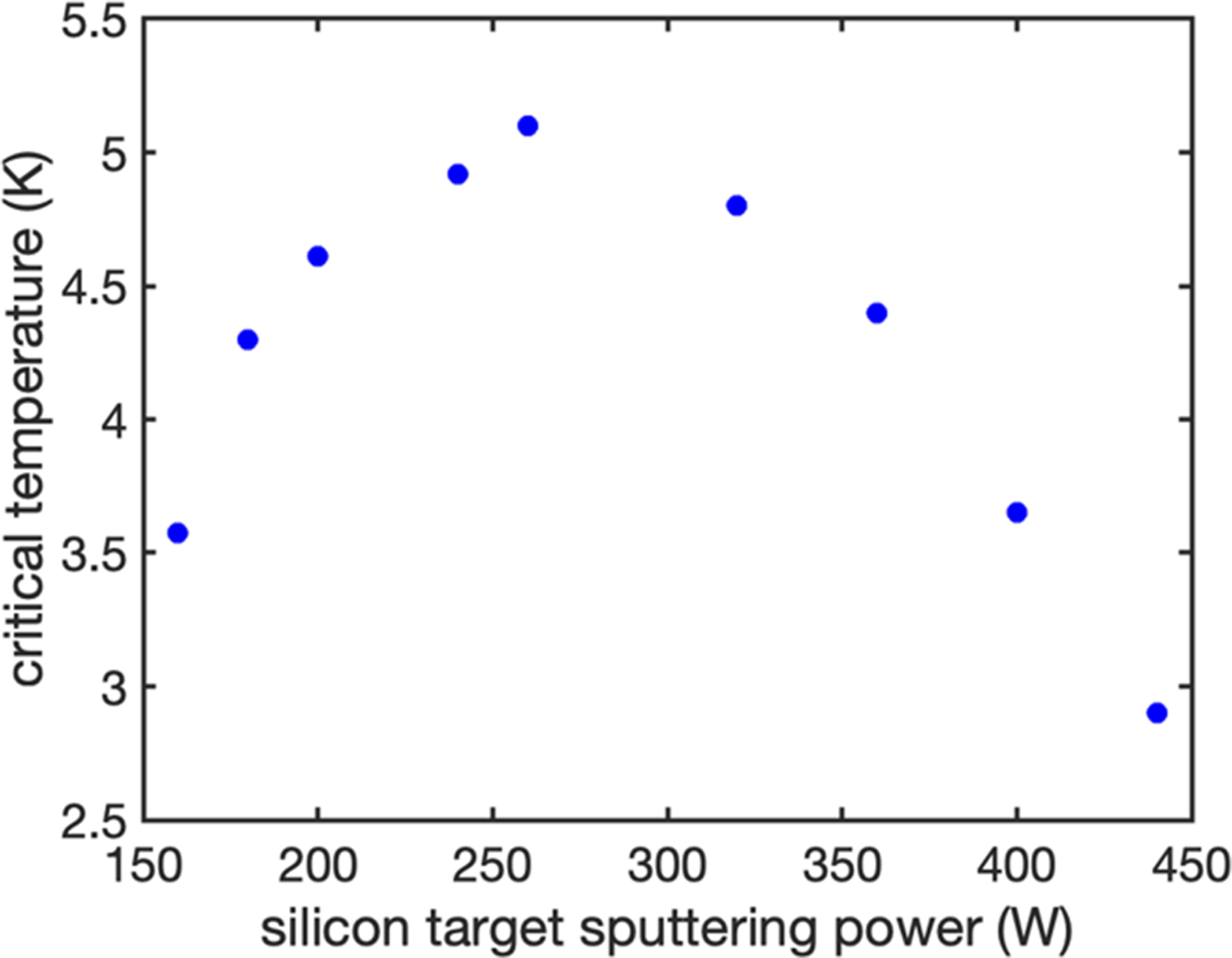
Superconducting transition temperature (*T*_c_) as a function of sputtering power on the silicon target during co-sputtering of the WSi film. The W sputtering power is fixed at 100 W. Measurements were performed on bulk films with thickness greater than or equal to 50 nm.

As outlined above, the most promising approach to lowering the energy threshold is to increase the resistivity of the superconducting film, which is typically achieved through the variation of the stoichiometry. For amorphous WSi, *T*_c_ is not strongly dependent on stoichiometry as shown in Fig. 1, which allows a significant variation in resistivity while maintaining a fixed operating temperature of the system. In addition, the films remain amorphous over a wide range of sputtering parameters and stoichiometries,^27^ as verified by x-ray diffraction measurements for the samples presented here.

## II. EXPERIMENT

**FIG. 2.**
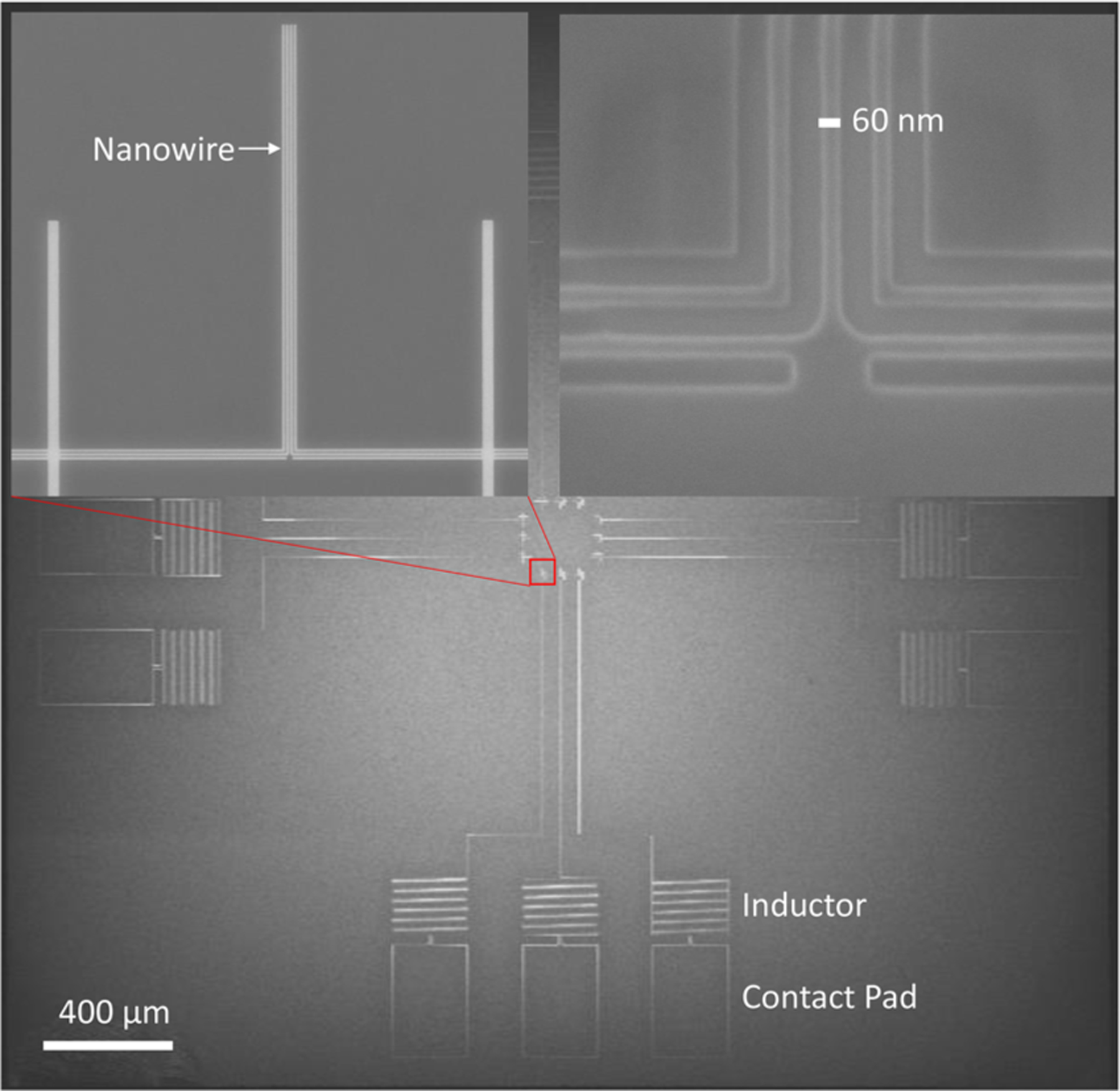
Scanning electron micrographs of the chip layout.

We fabricated detectors from high-resistivity WSi films using a Si-rich stoichiometry. The films are co-sputtered from separate W and Si targets, allowing the composition of the films to be tuned by adjusting the relative sputtering powers. Sputtering is performed with the substrate held at room temperature. Typical sputtering powers for the W and Si targets are 100 and 180 W, respectively, which in our deposition system results in a silicon content of 15 at. %. We increased the Si sputtering power from 180 to 260 W while maintaining the W sputtering power at 100 W. This resulted in a silicon composition of 35% ± 7% as estimated by secondary ion mass spectroscopy (SIMS). The film thickness was 3.2 nm, and the superconducting transition temperature was 3.1 K. Note that the *T*_c_ value of the thin film is significantly lower than that shown in Fig. 1 due to the decrease in *T*_*c*_ with film thickness. Each detector is a single 10 *μ*m-long nanowire instead of a large-area meander in order to reduce the probability of fabrication defects or constrictions along the length of the wire for the relatively narrow widths investigated—50 and 70 nm. A 2 *μ*H inductance consisting of a 10 *μ*m-wide meandering wire was patterned into the WSi film in series with the nanowire to prevent latching to the non-superconducting state.^28^ The scanning electron micrographs of the chip layout are shown in Fig. 2. The small active area of the device prevents it from achieving a high coupling efficiency in mid-infrared applications. Nevertheless, it serves as a well-controlled experiment to study the optimum nanowire material and geometry to achieve the maximum intrinsic detection efficiency.

**FIG. 3.**
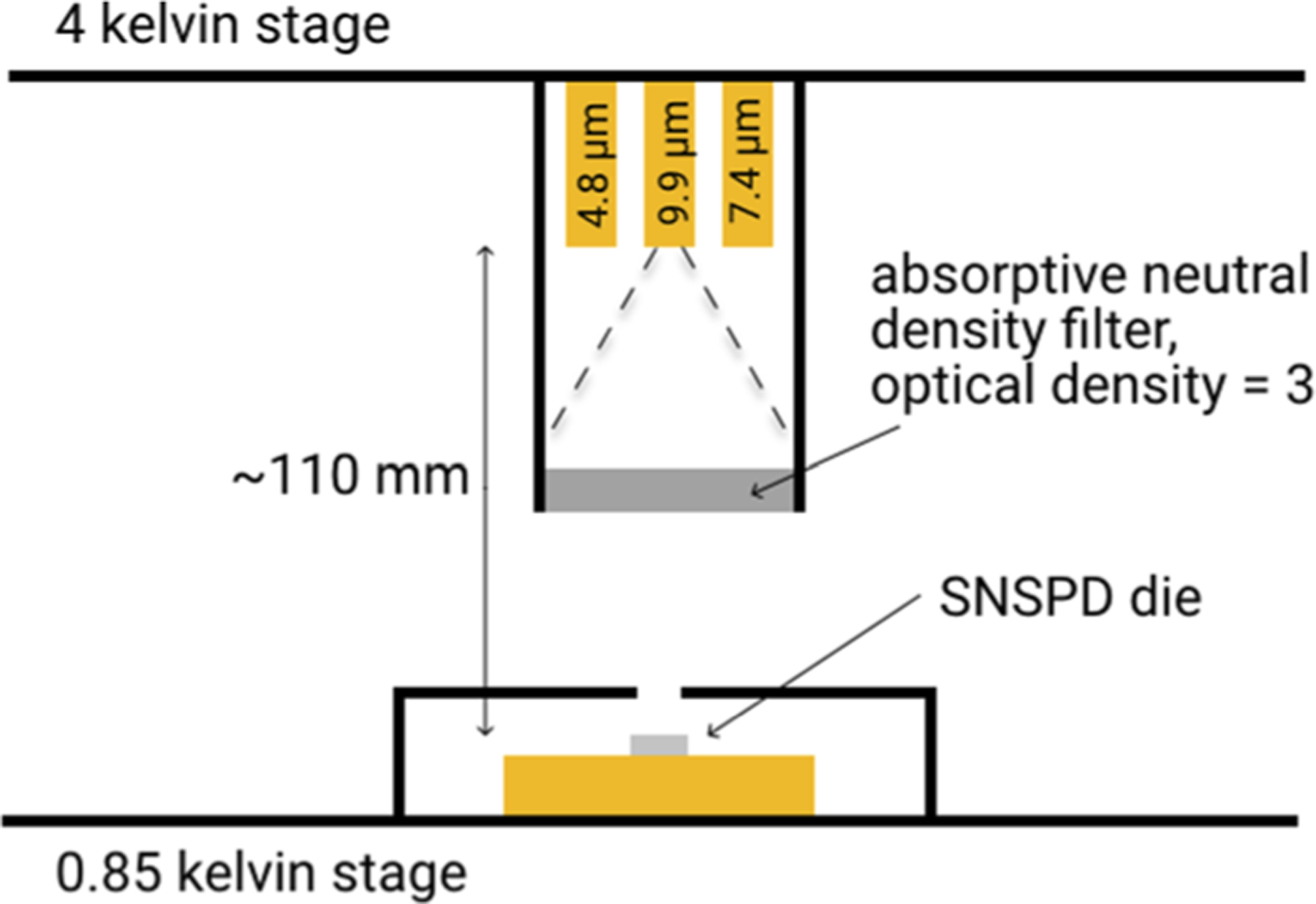
Quantum cascade laser (QCL) setup inside the cryostat, flood illuminating the sample. The divergence of the lasers is in the range of 50°–60° (FWHM), creating a significant amount of geometric attenuation, in addition to the neutral density filter (Edmund Optics No. 12–017).

The SNSPDs were measured at a temperature of 0.85 K and flood-illuminated using various quantum cascade lasers (QCLs) also mounted inside the cryostat at the 4 K stage. Figure 3 shows the measurement setup. The three QCLs (4.8, 7.4, and 9.9 *μ*m) are mounted side by side such that it is possible to study the response to different wavelengths during the same cooldown of the cryostat. The average full-width at half maximum (FWHM) divergence of the 4.8 and 7.4 *μ*m lasers was ∼53^°^ and 60^°^, respectively, and both QCLs were fabricated at the Jet Propulsion Laboratory (JPL).^29,30^ The divergence of the 9.9 *μ*m laser (Alpes Lasers^31^) was not directly measured but is expected to be similar to the other lasers in the vertical direction and ~40° in the horizontal direction. The QCLs were operated with 260 *μ*s pulses and a 4% duty cycle to minimize the heat load at the 4 K stage. To collect the photon count rate (PCR) curves, a 200 *μ*s gate was applied on a counter, synchronized with the middle of the laser pulses. The number of photons impinging on the detector in a single 260 *μ*s laser pulse was on the order of 100, which ensured that the detector was far from saturation, since its recovery time is >1000 times faster than the duration of the laser pulse. The background count rate (BCR) was collected by synchronizing the gate with the period when the laser is turned off. The signal from the SNSPD was amplified with a cryogenic amplifier (Cosmic Microwave Technology CIT-LF1^31^) operating at the 4 K stage, with a gain of 45 dB, a bandwidth of 1.5 GHz, and a noise temperature of less than 6 K. An additional room temperature amplifier with a gain of 25 dB and a bandwidth of 500 MHz was used, followed by a 120 MHz low-pass filter. This readout scheme enabled pulses to be read out while biased with currents as low as 200 nA.

**FIG. 4.**
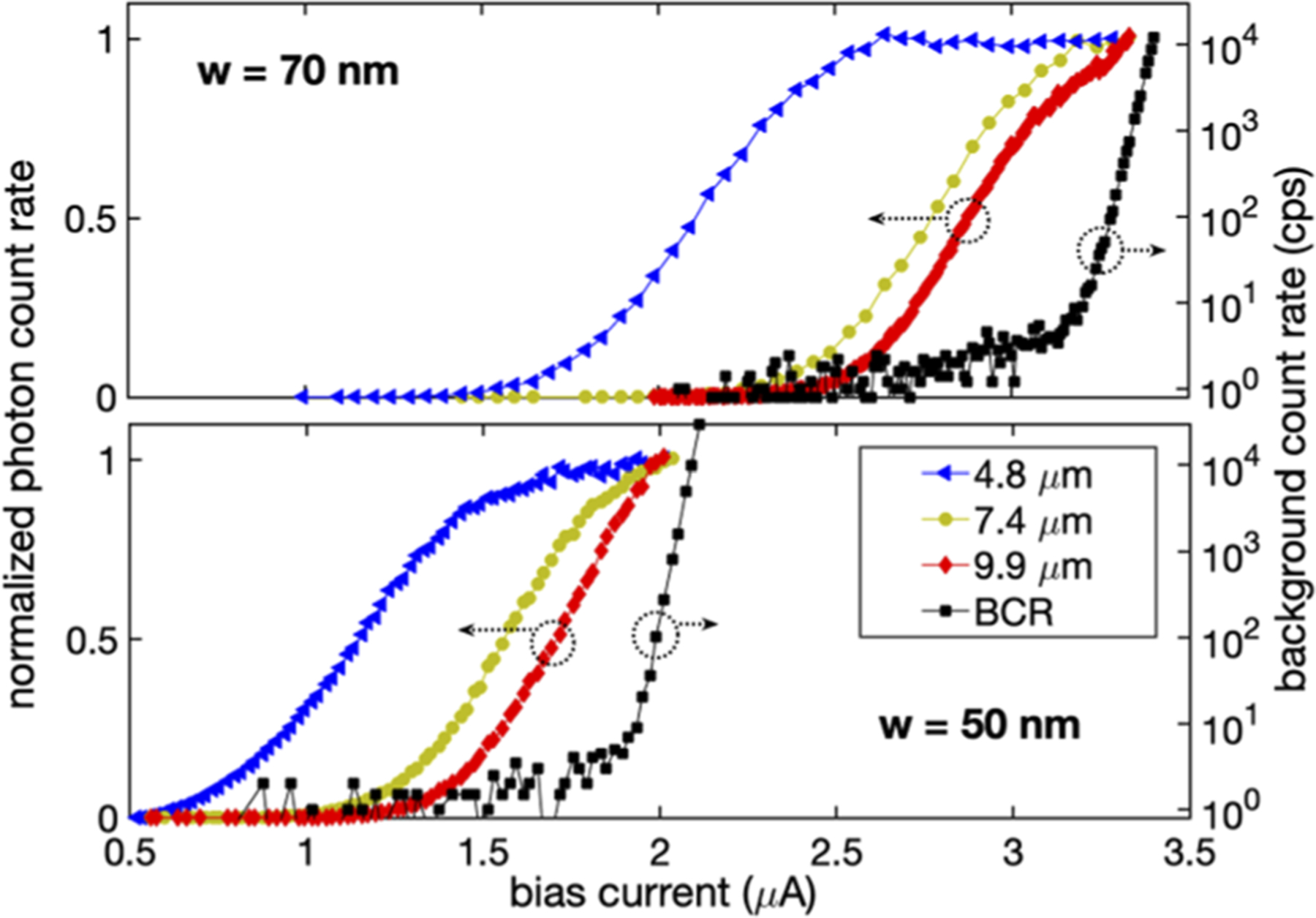
Normalized photon count rate vs bias current curves for SNSPDs fabricated from a WSi film with a silicon content of 35% ± 7% [the sputtering power on the silicon target increased from 180 to 260 W, compared to the material typically used for near-infrared (NIR) devices]. PCR curves are shown for two different nanowire widths (50 and 70 nm), and measurements were obtained at three wave-lengths (4.8, 7.4, and 9.9 *μ*m) at an operating temperature of 0.85 K. Black squares correspond to the measurement of the background count rates.

Figure 4 shows normalized PCR vs bias current curves for SNSPDs fabricated with a film deposited with a sputtering power on the silicon target of 260 W. PCR curves are shown for two different nanowire widths (50 and 70 nm), and measurements were obtained at three wavelengths (4.8, 7.4, and 9.9 *μ*m). The presence of a saturated plateau implies unity internal detection efficiency, where each absorbed photon leads to an electrical pulse. As shown in Fig. 4, the 70 nm-wide nanowire exhibits a clear inflection point in the PCR curve even for 9.9 *μ*m photons, which means that it is operating near the saturated internal detection efficiency point. The less-pronounced inflection point (see the supplementary material for PCR finite difference curves, to aid comparison) for the 50 nm wires is likely due to the presence of a fabrication defect such as a constriction in the width of the nanowire.

**FIG. 5.**
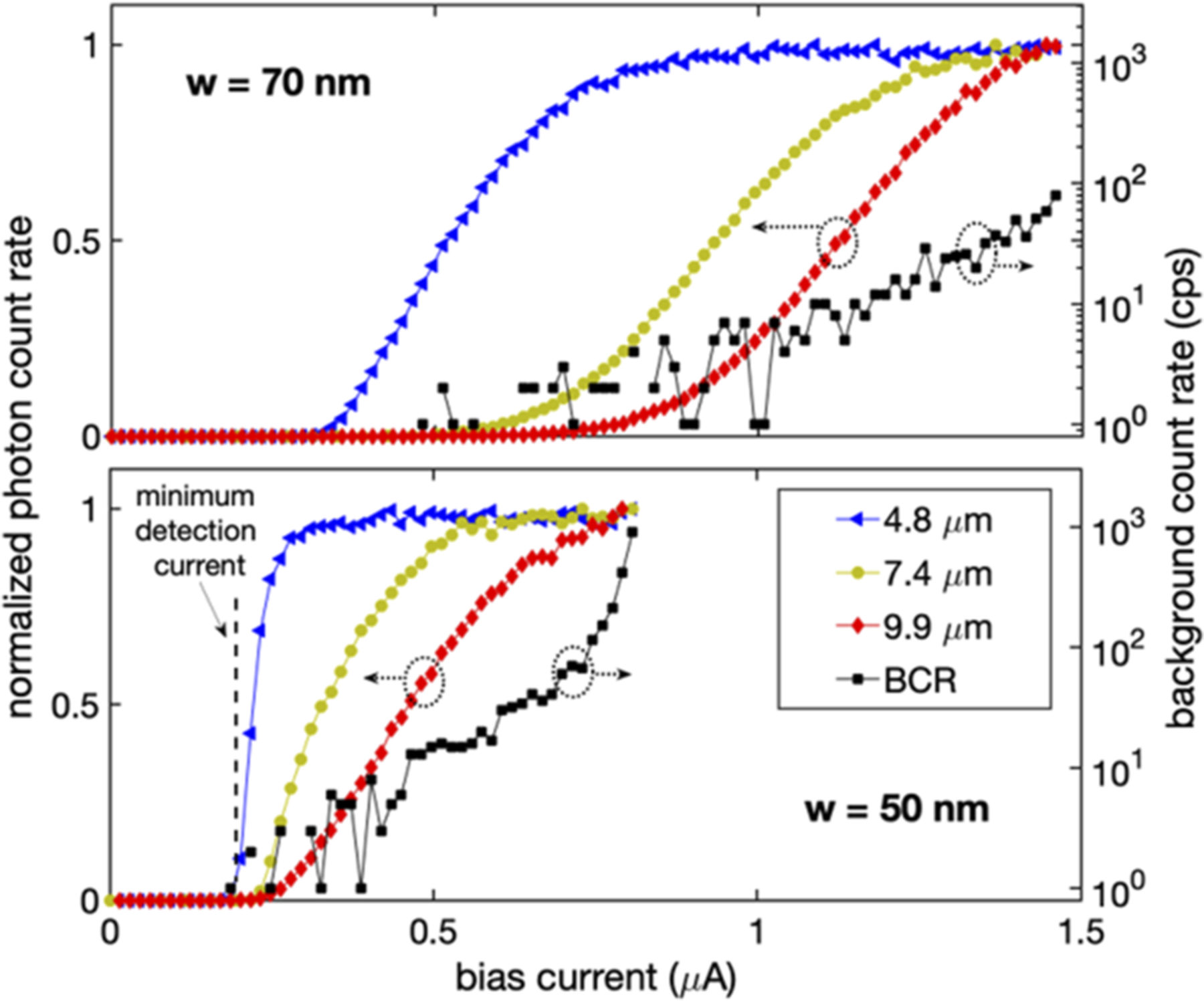
Normalized photon count rate vs bias current curves for SNSPDs fabricated from a WSi film with a further increase in the silicon content to 48% ± 10% (the sputtering power on the silicon target increased from 260 to 320 W compared to the devices in Fig. 4). Two different nanowire widths (50 and 70 nm) are presented, and measurements were obtained at three wavelengths (4.8, 7.4, and 9.9 *μ*m) at an operating temperature of 0.85 K. Black squares correspond to the measurement of the background count rates. The switching currents are lower; however, the relative saturation of the internal efficiency is better for all wavelengths and nanowire geometries (see the supplementary material for further discussion).

In order to further explore the effect of film stoichiometry on energy sensitivity, we fabricated a second set of devices with an even higher sputtering power on the silicon target of 320 W. SIMS analysis indicated a silicon composition of 48% ± 10%. The film thickness was determined to be 2.6 nm, slightly thinner than the previous set of devices with the lower silicon sputtering power, with a marginally lower *T*_c_ value of 2.8 K compared to the previous film with a *T*_c_ value of 3.1 K. Figure 5 shows the PCR vs bias current curves and dark count measurements for two nanowires having widths of 70 and 50 nm and a length of 10 *μ*m. Both geometries show a significant improvement in saturation of the internal detection efficiency at all three wavelengths (see the supplementary material for discussion). Note that the shape of the 4.8 *μ*m PCR curve for the 50 nm wire is distorted at the lowest bias currents because the readout threshold was about 200 nA; hence, any pulses created at lower currents were missed by the counter. To verify that the emission from the 9.9 *μ*m laser was at the expected wavelength, on one cooldown, we included a 10.6 *μ*m bandpass filter with a 1.5 *μ*m width, which resulted in the same SNSPD response as with the filter omitted. During this study, we succeeded in yielding nanowires down to 25 nm widths, using WSi films with an expected silicon composition in the 15%–20% range, as used in previous work for near-infrared devices. PCR curves with 9.9 *μ*m light for the low silicon-content films did not exhibit an inflection point for nanowire widths between 25 and 50 nm, which further supports the use of high-resistivity films for mid-infrared devices.

## III. CONCLUSION

In conclusion, we have fabricated WSi SNSPDs with a near-saturated internal detection efficiency at mid-infrared wavelengths up to 10 *μ*m. Increasing film resistivity by tuning the film stoichiometry appears to be a promising approach to improving energy sensitivity. Demonstrating the saturated internal efficiency is the first important step to obtaining high system detection efficiency, followed by efficient design of an optical stack around the detector to enhance absorption^15^ and optimized self-aligned fiber coupling^32^ or free-space coupling.^16^ However, with both fiber coupling and free-space coupling, filtering black-body radiation from the environment presents a significant engineering challenge in the mid-infrared.

In some applications, the signal to be detected originates from a cryogenic environment, but it should be ensured that the coupling fiber or optics are also cooled,^5^ while for space astronomy, the telescope itself must be actively cooled.^2^ For applications where the signal originates from a room temperature environment, the level of required filtering depends on the wavelength of interest since the peak of 300 K black-body emission is at ~10 *μ*m. For shortwave infrared (SWIR) wavelengths (up to 3 *μ*m), it is conceivable to combine narrow-band interference filters together with a sufficient amount of material absorption in glass, such as BK7, to sufficiently reduce the background for single photon applications. For mid-wave infrared (MWIR) and long-wave infrared (LWIR) applications, cryogenic spectrometers^5^ will likely be required to sufficiently channelize the background.

While the initial data presented here appear promising, further work is required to investigate the scalability and yield of narrow wires on the order of 50–80 nm over larger areas, which will be required for kilopixel and larger arrays required for applications such as exoplanet spectroscopy and dark matter science. If larger-area nanowire meanders can be yielded, simulations show that an optical cavity consisting of a gold back-reflector combined with a germanium-based dielectric cavity could yield an efficiency of 55%, comparable to the efficiency of typical blocked impurity band detectors albeit with a significantly smaller bandwidth. The peak wave-length may be tuned within the 2–10 *μ*m wavelength range by adjusting the thicknesses of the germanium layers. With a more complex dielectric layer stack consisting of alternating layers of germanium and a low-index dielectric such as MgF_2_ or BaF_2_, the peak efficiency could be increased further, perhaps as high as 80%. Achieving higher than 80% efficiency will be a challenge due to the fill factor, which is limited for fabrication reasons to roughly 50% with these ultra-narrow nanowires.

Methods of efficiency calibration of SNSPDs in the mid-infrared are currently being developed at NIST using a cryogenic spectrometer and a reference BIB detector. Both hollow-core optical fibers and chalcogenide glass fibers are well-developed technologies, which can be used for detector coupling.

Although we did not measure these SNSPDs at temperatures lower than 0.85 K, we do not anticipate significant improvement in performance with regard to the dark count rate or switching current by operating at lower temperatures. Dark count rates as measured here are limited primarily by the blackbody background. However, one could conceivably improve mid-infrared sensitivity further by decreasing the superconducting transition temperature as discussed in the Introduction. In this case, the use of a dilution refrigerator to achieve a lower operating temperature may be beneficial.

## Supplementary Material

Supp1

## Data Availability

The data that support the findings of this study are available from the corresponding author upon reasonable request.
