# Supplementary material for: Single-photon detection in the mid-infrared up to 10 μm wavelength using tungsten silicide superconducting nanowire detectors
Source: APL Photonics. Author manuscript; Available in PMC 2023 Aug 24. (PMC10448953; doi:10.1063/5.0048049)
Supplement: Supp1 [file NIHMS1918327-supplement-Supp1.pdf]

Supplementary information: Single-photon detection in the mid-infrared up to 10 micron wavelength using tungsten silicide superconducting nanowire detectors

V. B. Verma,[1,a)] B. Korzh,[2,b)] A. B. Walter,[2] A. E. Lita,[1] R. M. Briggs,[2] M. Colangelo,[3] Y. Zhai,[1] E. E. Wollman,[2] A. D. Beyer,[2] J. P. Allmaras,[2] H. Vora,[1] D. Zhu,[3] E. Schmidt,[2] A. G. Kozorezov,[4] K. K. Berggren,[3] R. P. Mirin,[1] S. W. Nam,[1] and M. D. Shaw[2]

[1)] *National Institute of Standards and Technology, Boulder, CO, USA.*

[2)] *Jet Propulsion Laboratory, California Institute of Technology, 4800 Oak Grove Dr., Pasadena, CA, USA*

[3)] *Department of Electrical Engineering and Computer Science, Massachusetts Institute of Technology, Cambridge, MA, USA.*

[4)] *Department of Physics, Lancaster University, Lancaster, UK*

(Dated: 21 April 2021)

[a)] Electronic mail: verma@nist.gov

[b)] Electronic mail: bkorzh@jpl.nasa.gov

The exact shape of the photon count rate (PCR) curve depends on the details of the microscopic detection mechanism[1–4], which is still an ongoing topic of research for long wavelength devices. The profile of the PCR curve reflects the probability that the detector will lead to a detectable signal when operated at a specific bias current. By biasing the detector on the saturated plateau implies unity internal detection efficiency - where each absorbed photon leads to an electrical pulse. Biasing the detector below this plateau region implies the detector is operating in a probabilistic regime, where some photons absorption events will go undetected. A convenient way to empirically compare PCR curves, for material optimization purposes presented in the main text, is to look at the finite difference of the PCR curve, since this contains information about the width of the probabilistic region, as well as how close the detector is to the fully saturated region. The most important feature of a PCR curve is the presence of an inflection point/points or peak/peaks in the PCR finite difference versus bias current, see Fig. 3 of Ref.[1]. By observing the location of the PCR curve inflection point/points and width/widths of transitions, it is possible to probe the specific features of detection mechanism and make a relative comparison of the optimum material and geometry for long-wavelength photons. Since the saturated plateau implies zero rate-of-change of the internal detection efficiency versus bias current, observing how close the finite difference gets to zero, for bias currents larger than the inflection point, will indicate how close to maximum internal efficiency the detector is capable of operating. As shown in Figures 1 and 2, all PCR curves presented in the main text exhibit a clear inflection point. Although qualitative, this technique provides a useful relative comparison of the intrinsic detection efficiency for different detectors. Figure 2 shows that the higher silicon-content devices exhibit PCR finite differences which approach lower values after the inflection point for 9.9 μm photons, before they become unstable, which implies a higher internal detection efficiency for these devices. Instability in the finite difference curves can arise near the device switching currents due to effects such as afterpulsing[5], which are important topics of future study in these mid-infrared devices.

The full experimental setup for collection of PCR curves, described in the main text, is illustrated in Fig. 3.

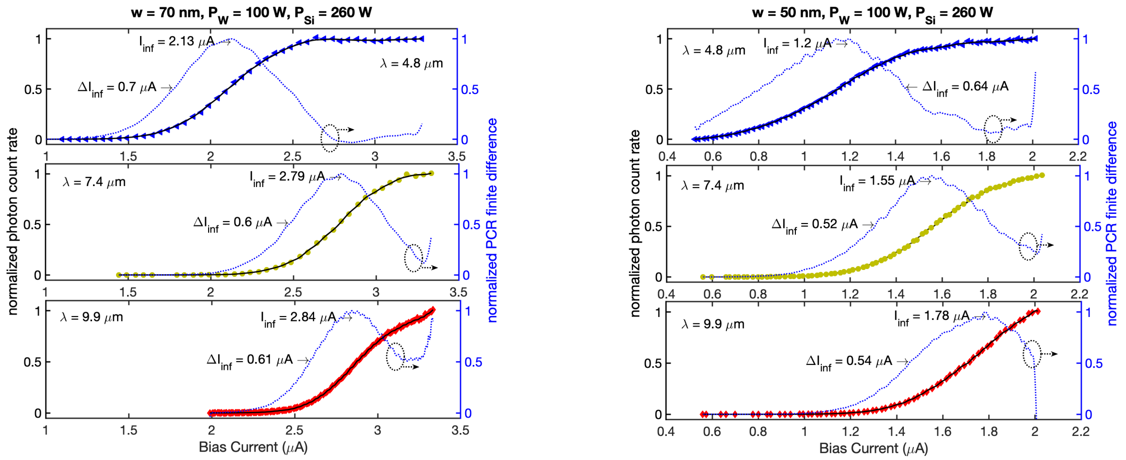

FIG. 1. Normalized photon count rate and finite difference curves for 70 nm-wide (left) and 50 nm-wide (right) nanowires fabricated from WSi films with 35% silicon content. The peak in the finite difference indicates the position of the PCR inflection point.

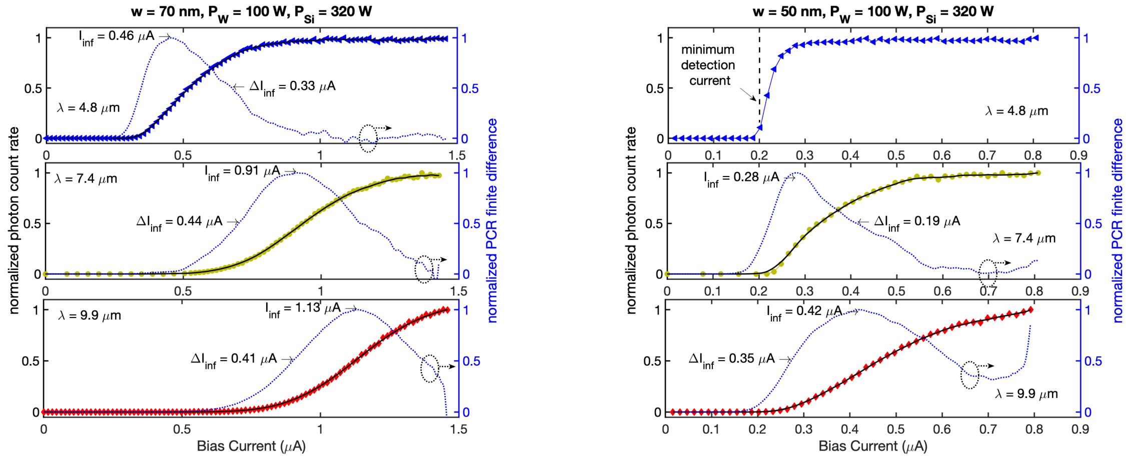

FIG. 2. Normalized photon count rate and finite difference curves for 70 nm-wide (left) and 50 nm-wide (right) nanowires fabricated from WSi films with 48% silicon content. Note that the finite difference for the 50 nm-wide device, illuminated with 4.8 µm light, is not presented since the PCR curve is distorted by the limit of the detection threshold at low bias currents currents (see main text), thus the width of the inflection point would be artificially narrower.

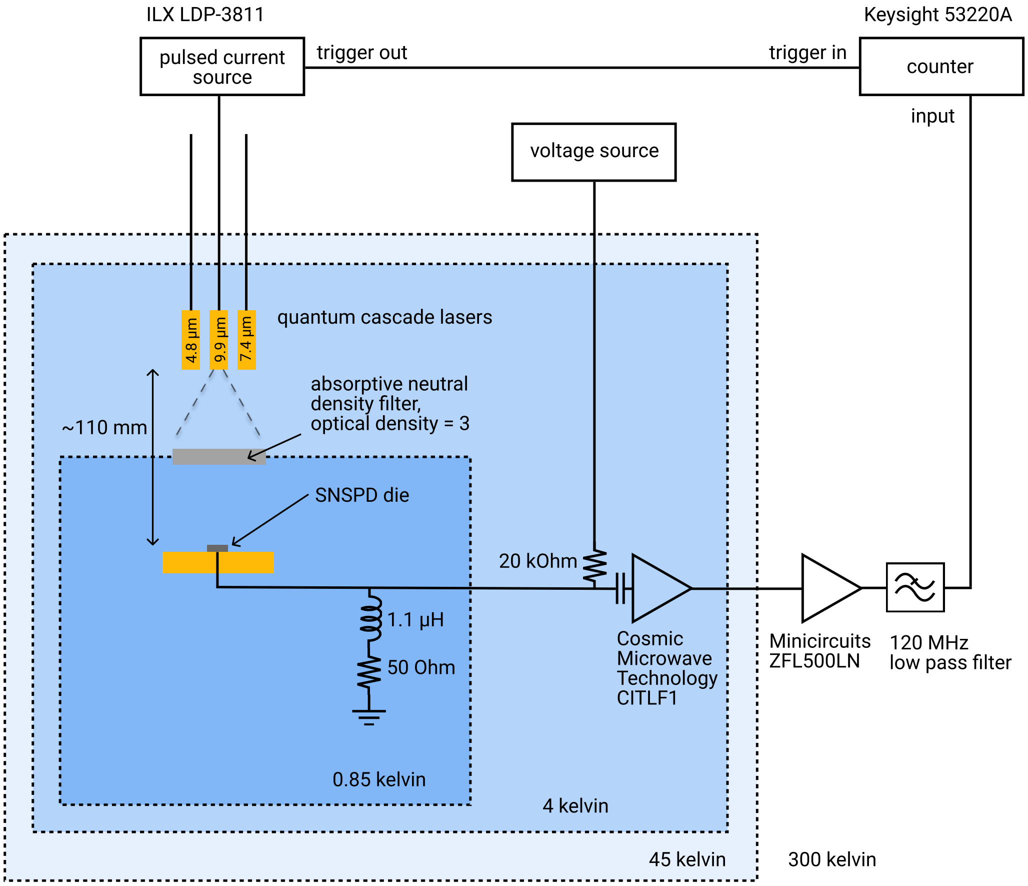

FIG. 3. Experimental setup for collection of SNSPD PCR curves with three different quantum cascade lasers, see main text for full description.

REFERENCES

[1]A. G. Kozorezov, C. Lambert, F. Marsili, M. J. Stevens, V. B. Verma, J. P. Allmaras, M. D. Shaw, R. P. Mirin, and S. W. Nam, "Fano fluctuations in superconducting-nanowire single-photon detectors," Phys. Rev. B **96**, 054507 (2017).

[2]D. Y. Vodolazov, "Single-photon detection by a dirty current-carrying superconducting strip based on the kinetic-equation approach," Phys. Rev. Applied **7**, 034014 (2017).

[3]J. P. Allmaras, A. G. Kozorezov, B. A. Korzh, K. K. Berggren, and M. D. Shaw, "Intrinsic Timing Jitter and Latency in Superconducting Nanowire Single-photon Detectors," Physical Review Applied **11**, 034062 (2019).

[4]J. P. Allmaras, *Modeling and Development of Superconducting Nanowire Single-Photon Detectors*, Ph.D. thesis, California Institute of Technology (2020).

[5]F. Marsili, F. Najafi, E. Dauler, R. J. Molnar, and K. K. Berggren, "Afterpulsing and instability in superconducting nanowire avalanche photodetectors," Applied Physics Letters **100**, 112601 (2012).
